# Identification of ASMTL-AS1 and LINC02604 lncRNAs as novel biomarkers for diagnosis of colorectal cancer

**DOI:** 10.1007/s00384-024-04692-x

**Published:** 2024-07-19

**Authors:** Fariba Shakeri, Parisa Mohamadynejad, Mehdi Moghanibashi

**Affiliations:** 1 Department of Biology, Faculty of Basic Sciences, Shahrekord Branch, Islamic Azad University, Shahrekord, Iran; 2 Department of Genetics, Faculty of Medicine, Kazerun Branch, Islamic Azad University, Kazerun, Iran

**Keywords:** Colorectal cancer, ASMTL-AS1, LINC02604, Bioinformatics, Biomarker

## Abstract

**Purpose:**

Colorectal cancer is one of the major leading causes of death worldwide, and available treatments for advanced colorectal cancer are not successful. Therefore, early detection of colorectal cancer is essential to improve patient survival, and biomarkers are potential tools to achieve this goal. Considering the key role of lncRNAs in cancers, the aim of this study is to identify lncRNAs involved in colorectal cancer as new potential prognosis biomarkers for CRC.

**Methods:**

In this observational study, gene expression data obtained from the TCGA database were analyzed, Identification of differentially expressed mRNAs, miRNAs, and lncRNAs was performed, and ceRNA network was drawn. Also, survival analysis of patients was performed in order to identify potential biomarkers related to the diagnosis and prognosis of colon cancer. After confirming the results using the GSE39582 dataset, the expression of target lncRNAs in colorectal tumor tissues was also investigated to confirm the bioinformatic data.

**Results:**

Analysis of the TCGA data showed that the expression of three lncRNAs—SNHG7, ASMTL-AS1, and LINC02604—that had the highest interaction with other miRNAs and mRNAs identified based on the ceRNA network was increased in colorectal cancer. Also, based on the ceRNA network, three microRNAs, hsa-let-7d-5p, hsa-mir-92a-3p, and hsa-mir-423-5p, and eight mRNAs, including CPA4, MSI2, RRM2, IGF2BP1, ONECUT2, HMGA1, SOX4, and SRM, were associated with all three mentioned lncRNAs, the expression of microRNAs was decreased and the expression of mRNAs was increased. By enrichment analysis, it was found that the target lncRNAs are involved in the processes of cell proliferation, apoptosis, and metastasis, indicating their importance in the development and malignancy of colorectal cancer. Furthermore, Kaplan–Meier analysis showed a significant increase in mortality in patients with higher expression levels of these lncRNAs. Analysis of the GSE39582 dataset, and real-time RT-PCR analysis, confirmed our bioinformatic results. Also, ROC analysis showed that SNHG7 was a relatively good promising biomarker (AUC = 0.73, *p* value = 0.02), while ASMTL-AS1 (AUC = 0.92, *p* value < 0.0001) and LINC02604 (AUC = 1.00, *p* value < 0.0001) emerged as excellent diagnostic biomarkers in colorectal cancer.

**Conclusion:**

It seems that increased expression of lncRNAs ASMTL-AS1 and LINC02604 can serve as molecular biomarkers for CRC, possibly through the sponge hsa-let-7d-5p, hsa-mir-92a-3p, and hsa-mir-423 5p, which increases target mRNAs, which are effective in the carcinogenesis process.

**Supplementary Information:**

The online version contains supplementary material available at 10.1007/s00384-024-04692-x.

## Introduction

Colorectal cancer is one of the leading causes of death worldwide [1]. Despite extensive efforts in developing diagnostic and prognostic methods, a significant proportion of patients are diagnosed in advanced stages, resulting in unsatisfactory treatment outcomes [2].

Although various environmental and genetic risk factors have been identified in relation to colorectal cancer [3], the exact molecular mechanisms involved in its development remain unclear. Therefore, it is crucial to gain a comprehensive understanding of the molecular mechanisms underlying colorectal cancer and discover biomarkers for early diagnosis.

Recent studies have uncovered significant findings regarding the essential role of long non-coding RNAs (lncRNAs) in various aspects of cell biology, including cell proliferation, apoptosis, metastasis, and treatment resistance. These molecules have the potential to contribute to the occurrence and progression of different types of tumors, including colorectal cancer [4–7]. LncRNAs are RNA molecules that exceed 200 nucleotides in length and lack the ability to encode proteins [7, 8]. However, they actively participate in the gene expression network through diverse mechanisms, such as miRNA sequestration, histone and chromatin modification, and protein translation [9, 10].

Emerging evidence suggests that the aberrant expression of lncRNAs may play a crucial role in the initiation and progression of colorectal cancer. As a result, these lncRNAs have been proposed as biomarkers and potential therapeutic targets for the diagnosis and management of the disease [11, 12]. Therefore, the identification of these lncRNAs holds promise for aiding in the early diagnosis and treatment of colorectal cancer.

In recent years, the development of high-throughput technologies, such as microarray and next-generation sequencing, has enabled the identification of numerous key genes associated with colorectal cancer that may contribute to its initiation and development [13].

Considering the pivotal role of lncRNAs as vital biomarkers in cancer, the present study was conducted with the aim of identifying lncRNAs involved in colorectal cancer. For this purpose, gene expression data obtained from TCGA were analyzed and ceRNA network was drawn. Also, survival analysis of patients was performed in order to identify potential biomarkers related to the diagnosis and prognosis of colon cancer. Next, the results of TCGA analysis were analyzed in the GSE39582 dataset from the GEO database, and finally, in order to confirm the results of bioinformatics analysis, the expression of target lncRNAs in tumor and healthy colorectal tissues was also investigated, and for the first time, two new biomarkers were introduced for the diagnosis of colorectal disease.

## Materials and methods

In this section, we used the STREGA reporting guidelines [14].

### Data source

To identify mRNAs, miRNAs, and lncRNAs associated with the development and pathogenesis of colorectal cancer, the TCGA data was utilized. The raw transcriptomic data of colorectal cancer (TCGA-COAD) was downloaded in HTseq-Counts format using the TCGAbiolinks package. Genes with zero or minimal expression were filtered out based on the criterion of less than 10 counts per million (CPM) in 70% of the samples, employing the edgeR package. Subsequently, the data underwent normalization using the TMM method (trimmed mean of M values) and the limma package. The resulting expression matrix was used for all subsequent analyses, comprising 480 tumor samples and 41 normal samples for mRNAs and lncRNAs, as well as 457 tumor samples and 8 normal samples for miRNAs. Additionally, the most recent clinical information was downloaded for all samples and incorporated into the analysis process.

Also, the raw data from the GSE39582 dataset was obtained from the GEO database, consisting of 19 normal and 542 colorectal cancer samples (www.ncbi.nlm.nih.gov/geo). The data underwent preprocessing steps, including background correction, data normalization using the RMA (robust multichip average) method, and transformation to logarithmic mode base 2 using the limma package.

### Identification of differentially expressed mRNAs, miRNAs, and lncRNAs

The normalized expression matrix was utilized to identify mRNAs, miRNAs, and lncRNAs that displayed significant expression changes in colorectal cancer samples compared to normal samples. The linear model method was employed to assess the expression differences between the groups, and a criterion of |logFC|> 1 and FDR < 0.01 was applied to select genes. Gene lists and information for mRNAs and lncRNAs were extracted using the Bio Mart tool. Furthermore, the expression changes were visualized using the Enhanced Volcano package through a volcano plot.

### Construction of the ceRNA network

The genes displaying significant expression differences were used to construct the ceRNA network. To investigate the interaction between miRNAs and mRNAs, the mirwalk database (http://mirwalk.umm.uni-heidelberg.de) and the mirTarBase database (http://miRTarBase.cuhk.edu.cn/) were employed. Only miRNA-mRNA pairs confirmed by both databases were selected.

Furthermore, the DIANA-LncBase v3 database (www.microrna.gr/LncBase) was utilized to explore miRNA-lncRNA interactions. The selection criteria included validation type = direct, miRNA Confidence level = high, and species = human. Subsequently, miRNA-mRNA and miRNA-lncRNA pairs were chosen based on their expression differences in colorectal cancer, meeting the criteria of |logFC|> 1 and FDR < 0.01 from the previous steps.

Finally, the data were visualized in Cytoscape software to represent the ceRNA network. The criterion of degree > 12 was used to identify the important lncRNAs within the ceRNA network.

### Preprocessing of clinical and prognosis data

Colorectal clinical data from TCGA-COAD were used to investigate the relationship between gene expression and patient prognosis. Preprocessing of clinical data was performed by removing normal samples, samples from patients with survival of 1 day or missing data (NA), and those without tumor-related mortality status.

Next, the expression levels of all genes were extracted for the samples meeting the specified clinical criteria. Z-scores were calculated to standardize the expression of each gene across all samples. The relationship between gene expression and patient prognosis was examined using the univariate Cox regression test.

Furthermore, Kaplan–Meier analysis was employed to confirm the obtained results. The median expression of candidate genes in cancer samples served as the cutoff point. A log Rank *p* value of less than 0.05 was considered statistically significant.

### Quantitative RT-PCR

Thirty-two colorectal tumor tissues and also adjacent normal tissue samples, based on the diagnosis of the attending physician and confirmation of the pathology results, from the Iranian race, were obtained from the Cancer Research Center, Cancer Institute (Tehran University of Medical Sciences, Tehran, Iran). RNA extraction was performed using TRIzol reagent (Invitrogen). Subsequently, cDNA synthesis was carried out using the cDNA Synthesis Kit (YTA), and RT-qPCR was performed using the SYBR green master-mix kit (YTA) and Rotor-gene 6000.

The GAPDH gene was used as the reference gene to normalize the expression level. Each reaction was repeated three times to ensure accuracy. The results were analyzed using the 2^−∆Ct^ method, a commonly employed quantitative analysis approach for gene expression studies.

Using Beacon software, specific primers were designed for all isoforms of LncRNAs SNHG7, LINC02604, ASMTL-AS1, mRNAs CPA4, SRM, SOX4, and GAPDH gene based on the sequence of genes in NCBI. The primer sequences used were as follows:

SNHG7, forward: 5′TCTCCTCCCGGCCAGTTC3′, reverse: 5′GCACCCGGAGGCCAGCAG3′; LINC02604, forward: 5′GCTAGACCATTTTTGTGCC3′, reverse: 5′CTGAAGGGACAATGCAAAC3′; ASMTL-AS1, forward: 5′GTTTACAGACGCATTTCAGCC3′, reverse: 5′GCTATGGAGTGGCAGTTCTC3′; GAPDH, forward: 5′GCCAAAAGGGTCATCATCTCTCTGC3′, reverse: 5′GGTCACGAGTCCTTCCACGATAC3′; CPA4, forward: 5′GACAACCCTTGCTCCGAAGT3′, reverse: 5′AGTAGCTGTGCAGGTCGATG3′; SRM, forward: 5′CTTTGTGCTGCCCGAGTTTG3′, reverse: 5′GTAACACTTGGTTGGTGGGC3′; SOX4, forward: 5′GACTTCGAGTTTGCTCCCCT3′, reverse: 5′TAACTCGCCTTCTTGCTGGG3′

### Statistical analysis

All preprocessing and data analysis were performed using the R programming language (version 4.0.2). GraphPad Prism software (version 8) was utilized for graphing and visualization purposes. Expression differences were calculated using the linear model method, and the significance level between the groups was assessed through multiple hypothesis testing. A threshold of FDR < 0.01 was applied for all analyses.

The relationship between the expression of candidate genes and patient prognosis was investigated using the log Rank test. A log Rank *p* value below 0.05 was considered statistically significant. In addition, Cytoscape software (version 4) was employed to display the ceRNA network and visualize the association of genes with the identified lncRNAs.

## Results

### Differentially expressed genes in colorectal *cancer*

To identify key genes involved in the progression and development of colorectal cancer, the TCGA data was evaluated. Differential expression analysis was conducted to compare lncRNAs, mRNAs, and miRNAs between cancer samples and normal samples.

**Fig. 1:**
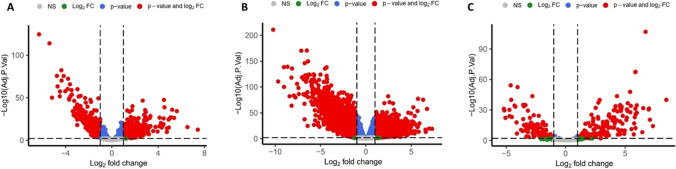
Volcano plots illustrating the expression levels of lncRNAs (**A**), mRNAs (**B**), and miRNAs (**C**). In these plots, red dots represent differentially expressed genes that fulfill both criteria of |logFC|> 1 and FDR < 0.01. Blue dots represent genes that do not meet the |logFC|> 1 criterion. Green dots represent genes that do not meet the FDR < 0.01 criterion, and gray dots represent genes that do not meet both the |logFC|> 1 and FDR < 0.01 criteria

The analysis revealed that among the examined genes, 403 lncRNAs, 3088 mRNAs, and 161 miRNAs exhibited increased expression in cancer samples compared to normal samples. Conversely, 280 lncRNAs, 3893 mRNAs, and 123 miRNAs showed decreased expression (Fig. 1) and may play important roles in the pathogenesis of colorectal cancer.

### Association of identified lncRNAs and mRNAs with the survival rate of patients

**Fig. 2:**
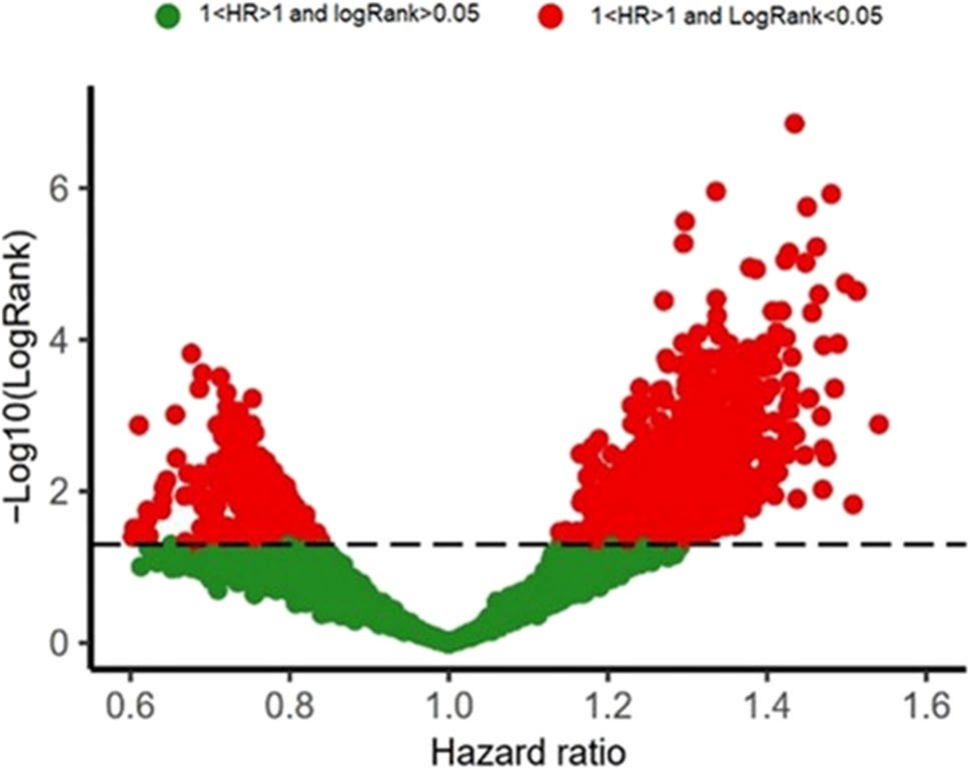
The volcano plot of the association of mRNAs with the survival rate of CRC patients

Our findings revealed that 1269 mRNAs were associated with poor prognosis (HR > 1, log Rank < 0.05), while 124 mRNAs were associated with good prognosis (HR < 1, log Rank < 0.05) (Fig. 2).

**Fig. 3:**
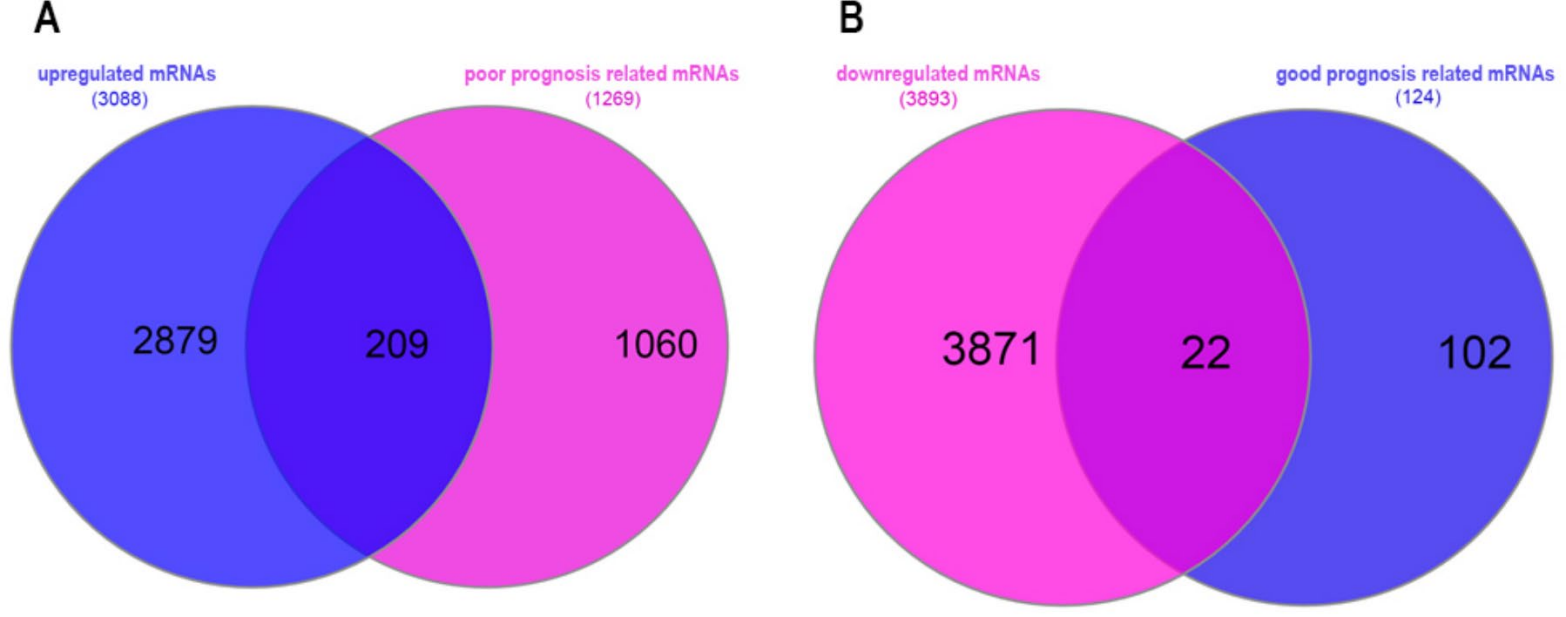
Intersection of differentially expressed mRNAs and survival-related mRNAs. **A** The intersected upregulated mRNAs associated with survival; **B** the intersected downregulated mRNAs associated with survival

Additionally, the results revealed that out of the differentially expressed mRNAs, 209 upregulated mRNAs were associated with poor prognosis of patients, whereas 22 downregulated mRNAs were associated with good prognosis of patients (Fig. 3).

**Fig. 4:**
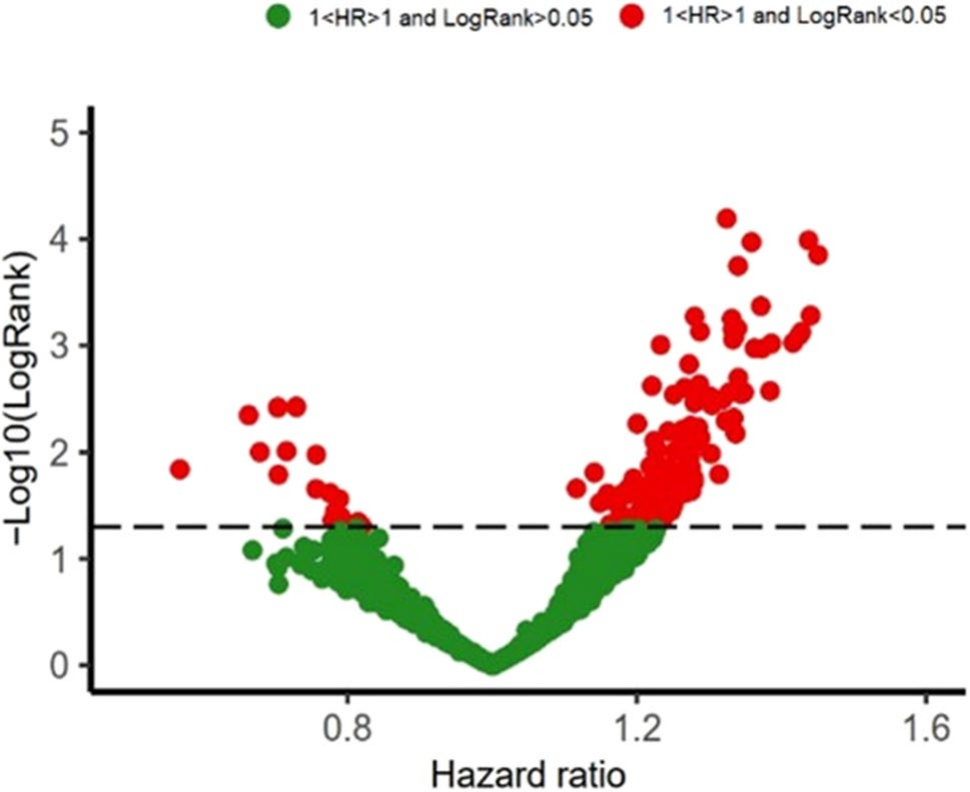
Volcano plot depicting the association of lncRNAs with the survival rate of colorectal cancer patients

Next, the association between lncRNAs and the survival rate of colorectal cancer (CRC) patients was investigated. The results revealed that 142 lncRNAs were associated with poor prognosis (HR > 1, log Rank < 0.05), while 17 lncRNAs were associated with good prognosis (HR < 1, log Rank < 0.05) (Fig. 4).

**Fig. 5:**
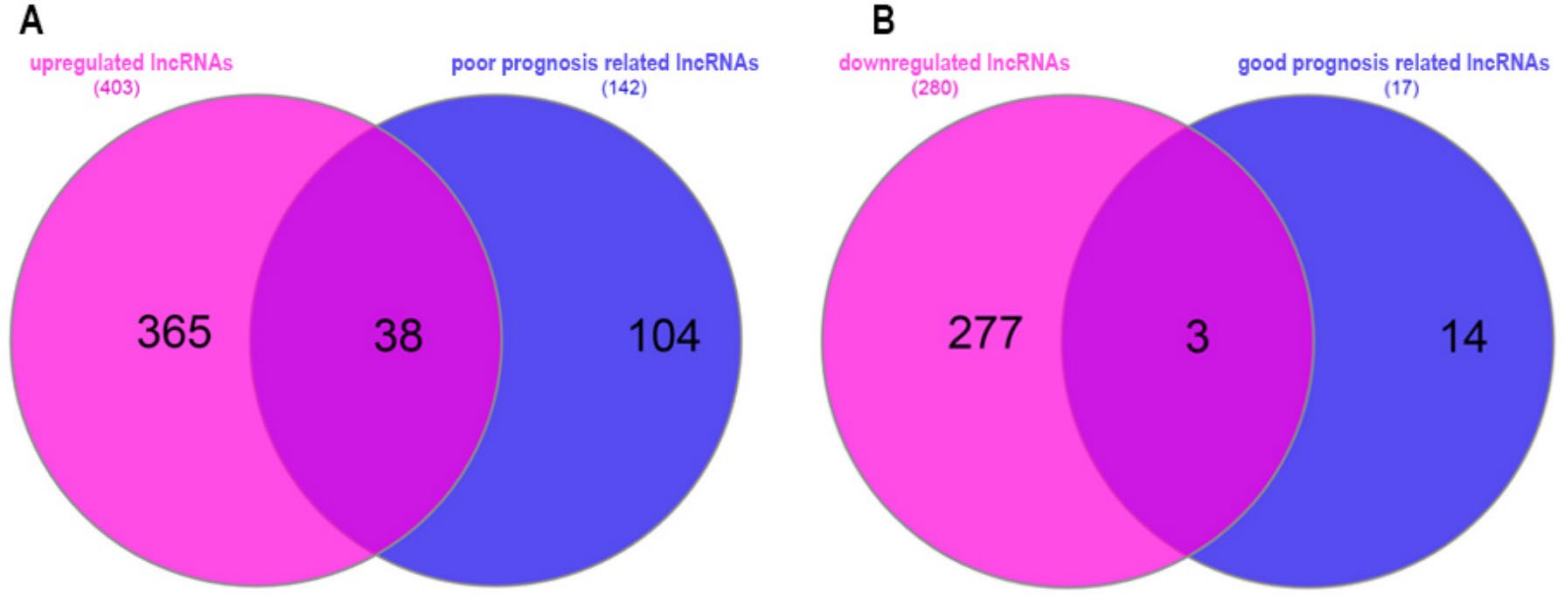
Intersection of differentially expressed lncRNAs and survival-related lncRNAs. **A** The intersected upregulated lncRNAs associated with survival; **B** the intersected downregulated lncRNAs associated with survival

Subsequently, an analysis was conducted to determine the overlap between differentially expressed lncRNAs and the identified survival-related lncRNAs. The findings revealed that 38 upregulated lncRNAs were associated with poor prognosis in patients, while 3 downregulated lncRNAs were associated with good prognosis (Fig. 5). These results suggest that the identified lncRNAs and mRNAs may play a significant role in the development of colorectal cancer.

### Identification of hub lncRNAs in the ceRNA network and their association with survival

**Fig. 6:**
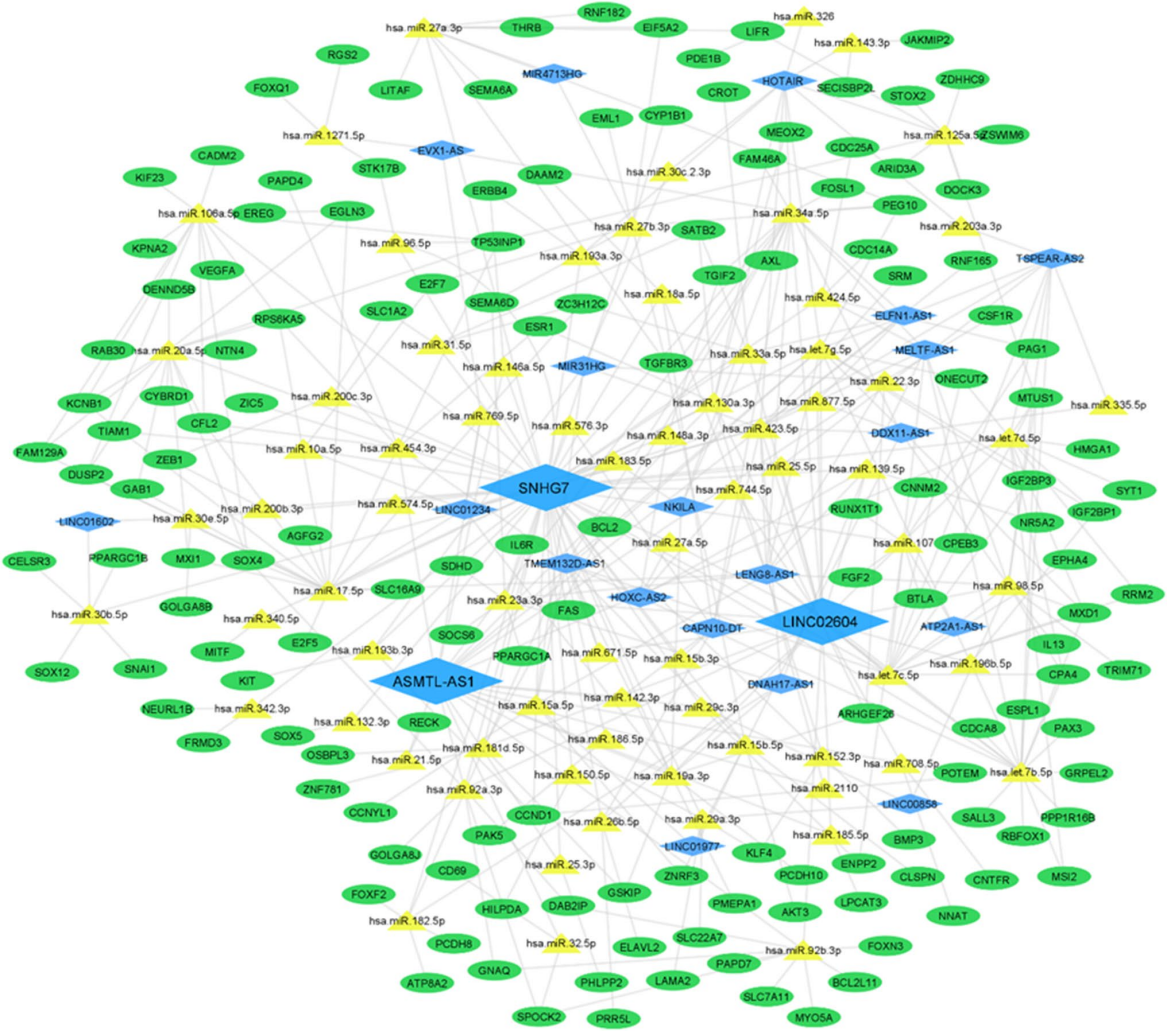
Illustration of the ceRNA network between all differentially expressed genes. In this network, blue diamonds represent lncRNAs, green ellipses represent mRNAs, and yellow triangles represent miRNAs

A ceRNA network was constructed using 161 upregulated and 123 downregulated miRNAs, along with differentially expressed mRNAs and lncRNAs associated with patient mortality. This network included 224 miRNA-mRNA pairs and 272 miRNA-lncRNA pairs, which were merged using Cytoscape. The results highlighted three lncRNAs, namely SNHG7, ASMTL-AS1, and LINC02604, which exhibited the most interactions with other identified miRNAs and mRNAs (Fig. 6).

**Fig. 7:**
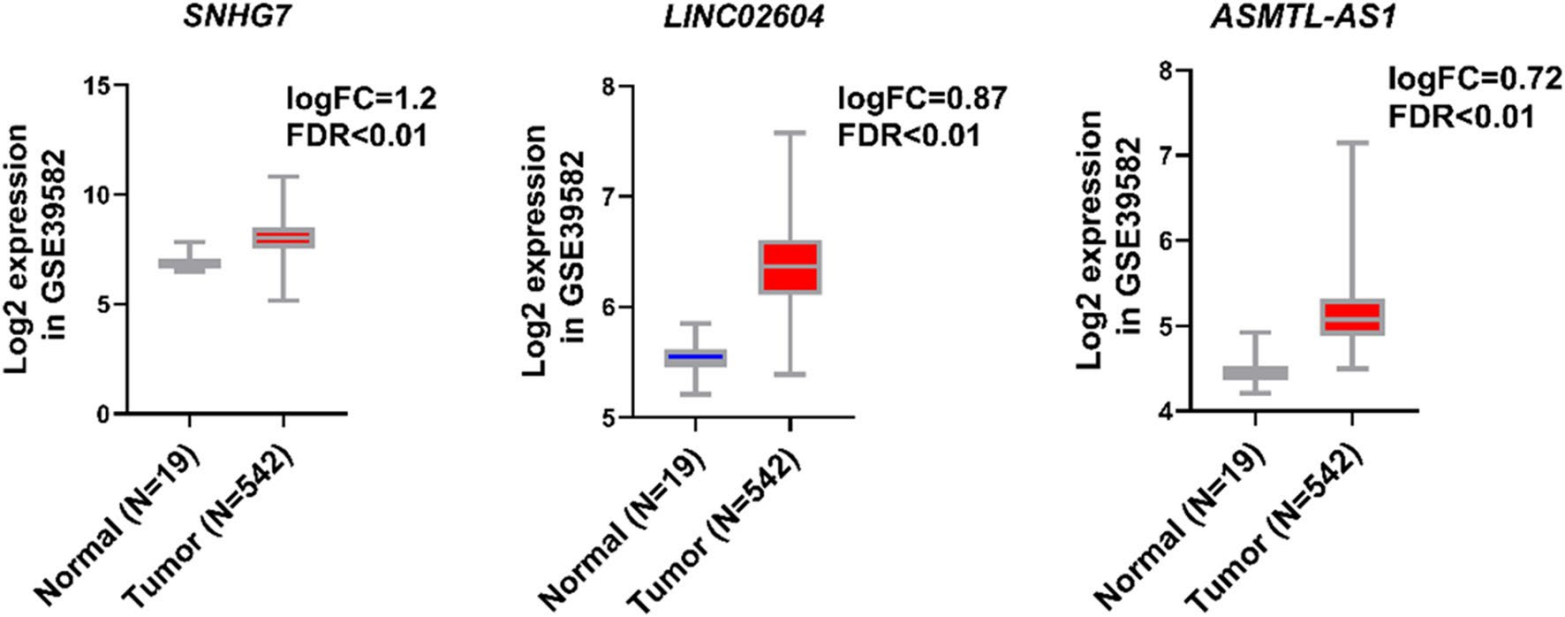
Comparison of the expression levels of SNHG7, ASMTL-AS1, and LINC02604 in the GSE39582 dataset

To validate our results obtained from TCGA, we assessed the expression levels of SNHG7, ASMTL-AS1, and LINC02604 in the GSE39582 dataset. The findings revealed a significant and substantial increase in the expression levels of SNHG7, ASMTL-AS1, and LINC02604 genes in cancer samples compared to normal samples (Fig. 7).

**Fig. 8:**
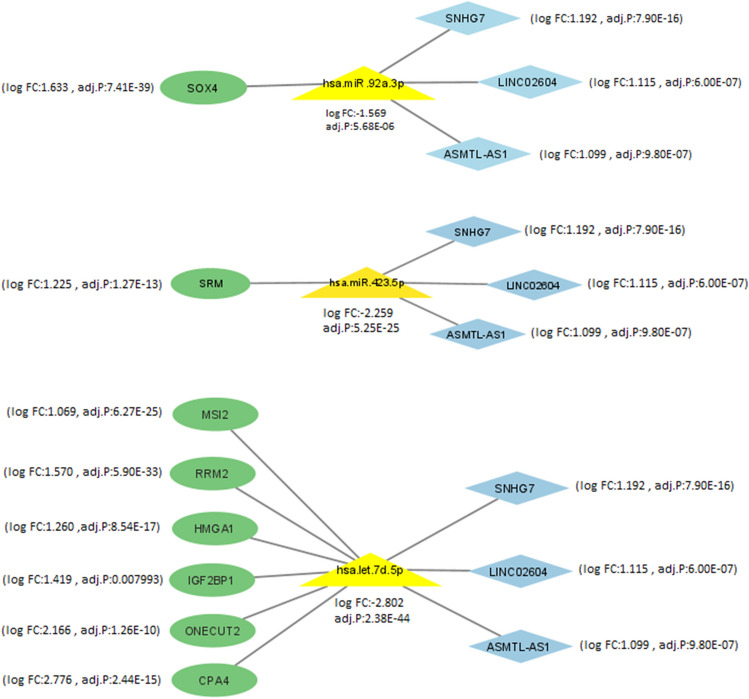
Association of target lncRNAs with hsa-let-7d-5p, hsa-mir-92a-3p, and hsa-mir-423-5p with the corresponding mRNAs

**Fig. 9:**
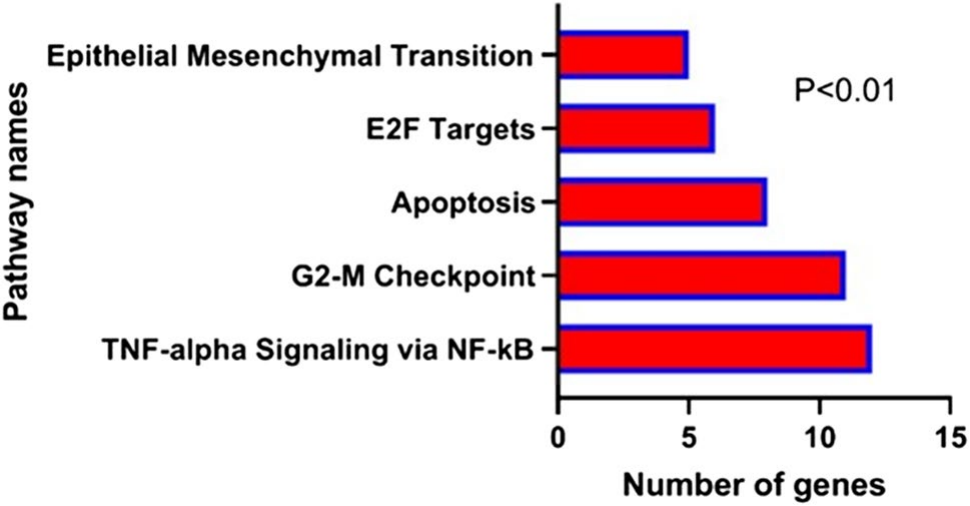
Enrichment analysis of all differentially expressed genes

Furthermore, hsa-let-7d-5p, hsa-mir-92a-3p, and hsa-mir-423-5p were found to be associated with each of the three mentioned lncRNAs, as well as with a total of eight mRNAs, including CPA4, MSI2, RRM2, IGF2BP1, ONECUT2, HMGA1, SOX4, and SRM (Fig. 8). Interestingly, our analysis revealed that the expression levels of the three miRNAs were decreased in tumor samples compared to normal samples, while the expression levels of the three lncRNAs and eight mRNAs were increased. Furthermore, the enrichment analysis of the genes in the ceRNA network revealed that a majority of these genes are involved in pathways related to cell proliferation, apoptosis, and metastasis (Fig. 9).

**Fig. 10:**
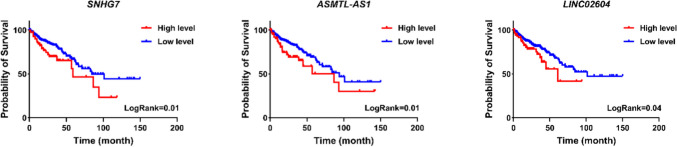
Association of expression levels of SNHG7, ASMTL-AS1, and LINC02604 with patients’ survival

To validate the obtained results, the relationship between the expression of SNHG7, ASMTL-AS1, and LINC02604 with the mortality rate of patients was investigated using Kaplan–Meier analysis. As depicted in Fig. 10, an increase in the expression levels of SNHG7, ASMTL-AS1, and LINC02604 in cancer samples was significantly associated with a higher mortality rate among patients.

These findings suggest that SNHG7, ASMTL-AS1, and LINC02604 may play a crucial role in the disease’s pathogenesis and the survival rate of patients.

### qRT-PCR

**Fig. 11:**
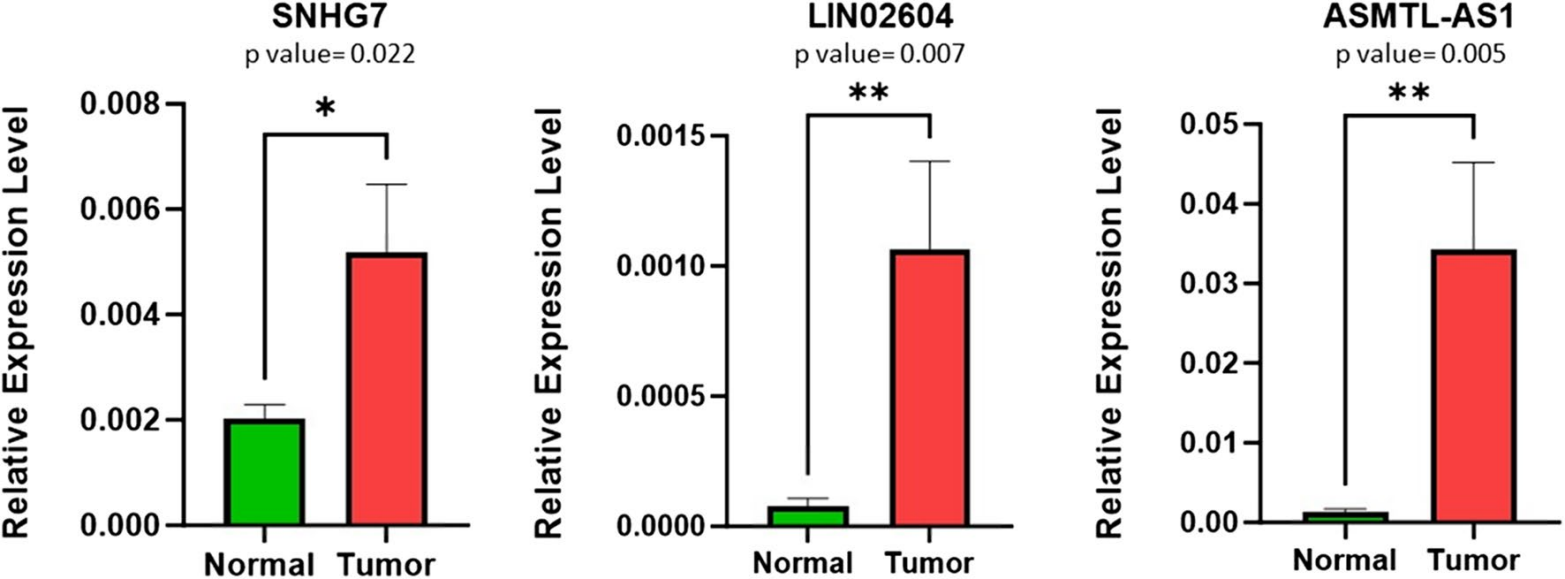
Comparison of the expression level of SNHG7, ASMTL-AS1, and LINC02604 lncRNAs in tumor tissues compared to adjusted normal tissues

Next, the expression levels of lncRNAs SNHG7, ASMTL-AS1, and LINC02604 were examined using qRT-PCR in 32 tumor and 32 adjacent normal colorectal tissues. The findings revealed an upregulation in the expression levels of all three lncRNAs in tumor tissues when compared to the adjacent healthy tissues (Fig. 11).

**Fig. 12:**
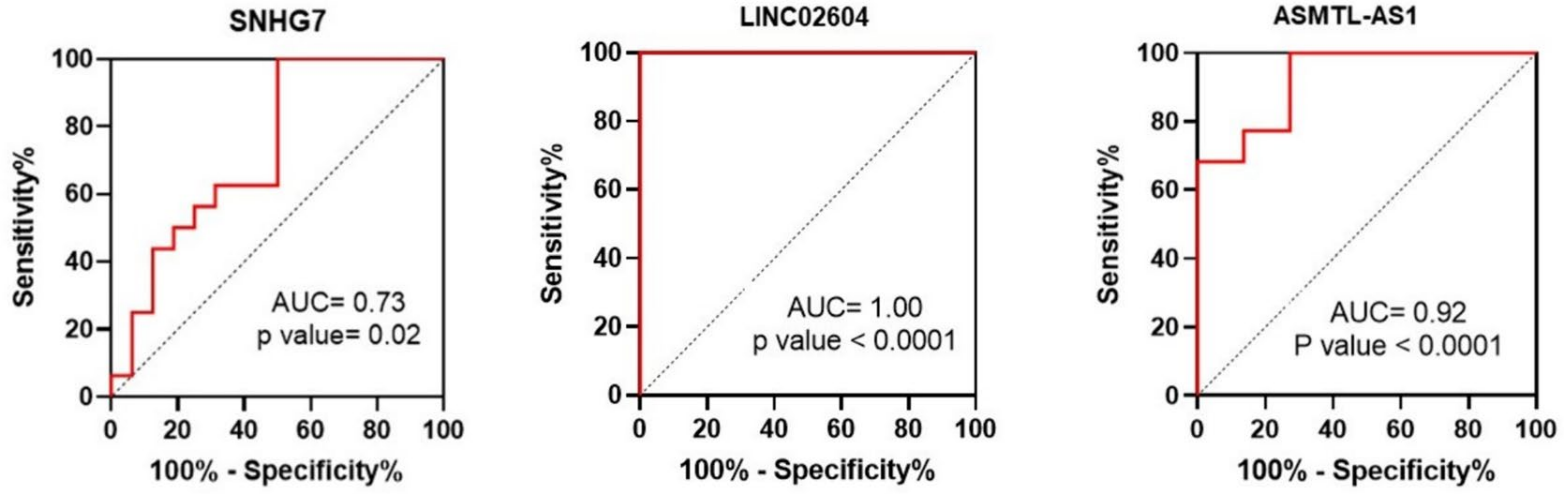
ROC curves of SNHG7, ASMTL-AS1, and LINC02604 lncRNAs

Furthermore, ROC analysis revealed that SNHG7 (AUC = 0.73, *p* value = 0.02) showed promise as a relatively good biomarker, while ASMTL-AS1 (AUC = 0.92, *p* value < 0.0001) and LINC02604 (AUC = 1.00, *p* value < 0.0001) emerged as excellent diagnostic biomarkers in colorectal cancer (Fig. 12).

**Fig. 13:**
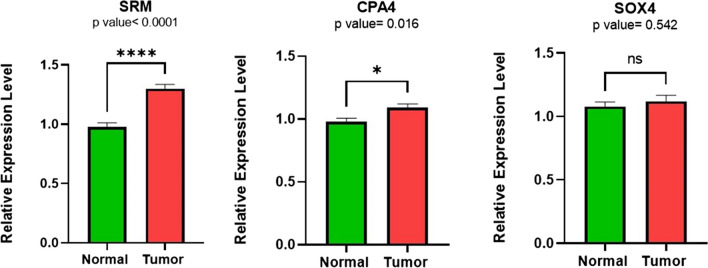
Comparison of the expression level of SRM, CPA4, and SOX4 mRNAs in tumor tissues compared to adjusted normal tissues

Finally, in the sub-networks related to target lncRNAs (Fig. 8), the expression levels of SOX4, SRM, and CPA4 (with the highest level of expression change based on bioinformatics analysis) mRNAs, in 32 tumor and 32 normal colorectal tissues, were investigated using qRT-PCR. Data analysis showed that the expression of SRM and CPA4 mRNAs in tumor tissue increased compared to the adjacent normal tissue, while the expression of SOX4 mRNA did not change (Fig. 13).

**Fig. 14:**
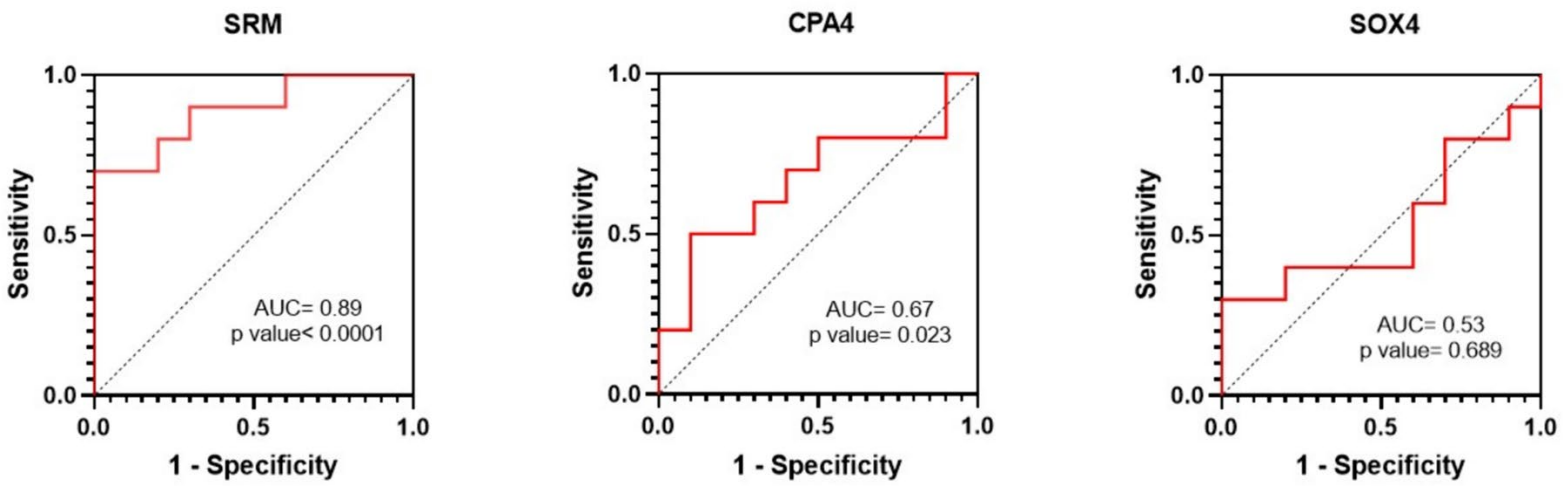
ROC curves of SRM, CPA4, and SOX4 mRNAs

Also, ROC analysis revealed that SRM (AUC = 0.89, *p* value < 0.0001) acts as an excellent diagnostic biomarker in colorectal cancer (Fig. 14).

## Discussion

A number of studies are currently looking for biomarkers to help achieve the goal of early detection of colorectal cancer, which is crucial to improving patient survival [15]. Long non-coding RNAs (lncRNAs) can significantly affect the development of tumors by controlling gene expression in a variety of cellular processes, including epigenetic modifications, mRNA stability, translation, alternative splicing, transcription, and miRNA regulation [16–18].

According to our results, the expression of three lncRNAs—SNHG7, ASMTL-AS1, and LINC02604—which have the highest number of interactions with other identified miRNAs and mRNAs, is increased in colorectal cancer and confirmed by analyzing the GSE39582 dataset from GEO and real-time RT-PCR analysis on colorectal tumor tissues compared to adjacent normal tissues.

Three lncRNAs were found to be involved in the processes of cell proliferation, apoptosis, and metastasis by enrichment analysis, suggesting their significance in the growth and malignancy of colorectal cancer. Additionally, Kaplan–Meier analysis indicated a significant increase in mortality in patients with higher expression levels of these lncRNAs.

In addition, based on bioinformatics data, a total of eight mRNAs, including CPA4, MSI2, RRM2, IGF2BP1, ONECUT2, HMGA1, SOX4, and SRM, were related to each of the three lncRNAs, as well as hsa-let-7d-5p, hsa-mir-92a-3p, and hsa-mir-423-5p. Considering the decrease in the expression of these miRNAs and the increase in the expression of target mRNAs, it seems that the mRNA/miRNA/LncRNA axes can be taken into consideration.

Recent studies have shown that SNHG7 acts as an oncogene, and its expression significantly increased in a wide range of carcinomas, including pancreatic cancer, thyroid cancer, bladder cancer, breast cancer, cervical cancer, gastric cancer, hepatocellular carcinoma, hypopharyngeal cancer, melanoma, and neuroblastoma [19]. It has been reported that SNHG7 acts as a ceRNA by sponging miR-34a, which controls the expression of the GALNT7 target gene and promotes the progression of colorectal cancer (CRC) via the PI3K/Akt/mTOR pathway [15]. Additionally, through the miR-216b sponge, SNHG7 can upregulate GALNT1 expression. This can result in oncogenic effects through the SNHG7/miR-216b/GALNT1 axis, and it has been proposed that targeting this axis could be a promising therapeutic approach for CRC [20].

The antisense acetyl serotonin O-methyltransferase 1 (ASMTL-AS1) long non-coding RNA was found recently at the Xp22.33 and Yp11.2 locus, and it plays a dual role in cancer. In breast cancer, ASMTL-AS1 expression is significantly downregulated, which triggers the Wnt/β-catenin signaling pathway via the miR-1228-3p/SOX17 axis [21]. However, in hepatocellular carcinoma, ASMTL-AS1 upregulation activates the YAP signaling pathway through the ASMTL-AS1/miR-1343-3p/LAMC1 axis, which results in a recurrence of the cancer or its metastasis [22]. Nevertheless, little is known about the expression and function of ASMTL-AS1 lncRNA in colorectal cancer; our study gives the first evidence of its oncogenic role in colorectal cancer.

In addition, there is not much information about the lncRNA LINC02604. According to Shi et al., in glioblastoma tumors, high expression of LINC02604 increases the probability of cell proliferation, invasion, and migration, which is associated with poor prognosis and decreased patient survival [23]. Also, in a bioinformatics study, Jing et al. identified LINC02604 as a potential biomarker to identify colon cancer patients who benefited from antitumor immunotherapy [24]. The results of both studies are in line with the results of the present study.

According to the ceRNA network identified in this study and the evaluation of target mRNAs of these lncRNAs, it can be suggested that target lncRNAs can exert these oncogenic effects through hsa-mir-423-5p/SRM or hsa-let-7d-5p/CPA4 axes. Also, other hsa-let-7d-5p/MSI2-RRM2-IGF2BP1-ONECUT2-HMGA1 axis should be investigated.

MiR-423-5p has been found to be downregulated in several tumors, including ovarian cancer, osteosarcoma, cervical cancer, and colon cancer [25]. In colon cancer, miR-423-5p as a tumor suppressor has been reported to induce cell apoptosis through caspase activation, and interestingly, it has been introduced as a biomarker for early detection of colon cancer [26]. Also, Let-7d, a member of the let-7 family, acts as a tumor suppressor in various cancers, including colorectal cancer [27], and hsa-let-7d-5p expression is significantly downregulated in CRC tumoral tissues compared to adjacent normal tissues [28, 29]. As a member of the miR-17–92 family, miR-92a-3p, as an oncogene or tumor suppressor gene [30], plays an important role in regulating cell viability, apoptosis, and metastasis of tumor cells [31, 32], and its dysregulation is associated with tumor progression and prognosis [33].

Carboxypeptidase A4 (CPA4) is a zinc-dependent metallocarboxypeptidase that is overexpressed in various cancer tissues, including colorectal cancer (CRC), which is in line with the results of the present study. Increased expression of CPA4 promotes the growth of CRC cells, while its knockdown results in decreased proliferation, arrest in the G1/S phase transition, and induction of apoptosis. Accordingly, CPA4 has been considered a prognostic factor or therapeutic target for CRC [34].

The level of several polyamines increases in various cancers such as skin, breast, colon, lung, and prostate cancer [35], which play an important role in cell proliferation, tumor invasion, and metastasis by modulating gene expression and signaling pathways [36, 37]. Spermidine synthase (SRM) is an enzyme involved in the biosynthesis of various polyamines, including putrescine, spermidine, and spermine [38]. In this regard, it has been reported that SRM gene expression is upregulated in colorectal cancer through the function of lncRNA ELFN1-AS1 and miR-423 sponge [39] which can be a confirmation of our results.

Also, the increase in the expression of MSI2, RRM2, IGF2BP1, ONECUT2, and HMGA1 mRNAs has been reported in different studies. The RRM2 gene has been introduced as an oncogene in several cancers, including CRC, so its increased expression is associated with advanced tumor grade, poor prognosis, and reduced patient survival, and its inhibition through the induction of cell apoptosis can be a potential therapeutic strategy for CRC [40–43].

Sex-determining region high mobility group box 4 (SOX4), as a transcription factor, regulates multiple signaling pathways, including PI3K, Wnt, and TGFβ, and promotes tumorigenesis by increasing cell survival, migration, invasion, and metastasis. Therefore, it is not surprising that the expression of SOX4 is increased in various malignancies, including breast, prostate, stomach, and colon cancer, and that it acts as an oncogene [44–46] which was not confirmed in the present study. It seems that the role of miR-92a-3p in colorectal cancer should be clearly defined.

The Musashi-2 (MSI-2) gene is overexpressed in various tumors, including colorectal cancer, and plays an important role as an oncogene in carcinogenesis and tumor progression [47]. Increased cytoplasmic expression of MSI-2 in CRC patients is associated with an unfavorable prognosis and has been proposed as a potential biomarker for CRC prognosis [48]. Increasing expression of transcription factor high mobility group protein A1 (HMGA1) can cause the development of cancer by regulating the transcription of targets of several biological pathways, which is associated with poor clinical results, distant metastasis, and advanced tumor stage in many cancers. HMGA1 expression has been reported to be increased in colorectal cancer and can serve as a diagnostic indicator for CRC [49].

Increased expression of the transcription factor one-cut domain 2 (ONECUT2) has been observed in various cancers, including colorectal cancer, which is associated with tumor growth, metastasis, chemoresistance, and poor prognosis and is considered a potential therapeutic target in CRC [50]. Finally, in limited adult tissues, increased expression of the insulin-like growth factor 2 mRNA-binding protein 1 (IGF2BP1) gene has been reported in cancers, including gastrointestinal cancers, lung cancer, melanoma, and colorectal cancer [51].

## Conclusion

The data of this research showed that the increased expression of target lncRNA, especially ASMTL-AS1 and LINC02604, can act as a biomarker in the identification of colorectal tumors. Based on bioinformatic data, it seems that SNHG7, ASMTL-AS1, and LINC02604 can act as molecular sponges, through the reduction of hsa-let-7d-5p and hsa-mir-423-5p, which leads to the increase of target mRNAs, specially RSM and CPA4 mRNAs which are related to the development and progression of colorectal cancer through the regulation of cell proliferation, apoptosis, invasion, metastasis, and response to treatment. Investigating the functions and interactions of these genes in a broader way can provide new insight into the underlying mechanisms of colorectal cancer and potentially develop therapeutic strategies.

## Supplementary Information

Below is the link to the electronic supplementary material.

Supplementary file1 (DOCX 28 KB)

## Data Availability

The datasets analyzed during the current study are available in Gene Expression Omnibus (GEO) (http://www.ncbi.nlm.nih.gov/projects/geo/) and *The Cancer Genome Atlas* (TCGA) (https://tcga-data.nci.nih.gov/).
